# Time fractional model of electro-osmotic Brinkman-type nanofluid with heat generation and chemical reaction effects: application in cleansing of contaminated water

**DOI:** 10.1038/s41598-021-03062-9

**Published:** 2021-12-22

**Authors:** Hussam Alrabaiah, Muhammad Bilal, Muhammad Altaf Khan, Taseer Muhammad, Endris Yimer Legas

**Affiliations:** 1 College of Engineering, Al Ain University, Al Ain, United Arab Emirates; 2 Department of Mathematics, Tafila Technical University, Tafila, Jordan; 3 Department of Mathematics, City University of Science and Information Technology, Peshawar, Pakistan; 4 Institute for Groundwater Studies, Faculty of Natural and Agricultural Sciences, University of the Free State, Bloemfontein, South Africa; 5 Department of Mathematics, College of Sciences, King Khalid University, Abha, 61413 Saudi Arabia; 6 Department of Mathematics, College of Natural Science, Wollo University, Dessie, Ethiopia

**Keywords:** Engineering, Materials science, Mathematics and computing, Nanoscience and technology

## Abstract

Drilling fluids execute a dominant role in the extraction of oil and gas from the land and rocks. To enhance the efficiency of drilling fluid, clay nanoparticulate has been utilized. The inclusion of clay nanomaterial to drilling fluids significantly elevate their viscosity and thermal conductivity. Therefore, the present investigation is focused on the analysis of time-fractional free convective electro-osmotic flow of Brinkman-type drilling nanofluid with clay nanoparticles. The heat generation and chemical reaction characteristics and influence of the transverse magnetic field have also been taken into an account. The local mathematical model is formulated in terms of coupled PDEs along with appropriate physical conditions. The dimensional governing equations have been non-dimensionalized by using relative similarity variables to encounter the units and reduce the variables. Further, the non-dimensional local model has been artificially converted to a generalized model by utilizing the definition of time-fractional Caputo–Fabrizio derivative with the exponential kernel. The graphical results are analyzed via computational software Mathematica, to study the flow behavior against inserted parameters. From graphical analysis it has been observed qualitatively that the velocity field has been raised against the greater magnitude of electro-osmosis parameter $Es$. Numerical table for Nusselt number is calculated from the obtained exact solutions. From the analysis 11.83% elevation in the rate of energy transition of drilling nanofluid has been reported in response of clay nanoparticles.

## Introduction

Due to multidimensional and phenomenal features, fractional calculus is growing rapidly day by day. Nowadays the implementation of fractional calculus is not limited to the problems of mathematics only but also contributing to solving the problems in many sectors like elasticity, chaos, diffusion, polymerization, etc. Fractional calculus is the extended and generalized version of classical calculus that contains the order of the derivative and integral in non-integer form. Fractional calculus is a very effective and efficient tool for the elaboration of heredity and the memory effect of the phenomena. In the last few years, remarkable development has been done by using fractional calculus^1–4^, such as wave propagation^5^, image processing^6^, modeling of cardiac tissue^7^, analysis of silver nanoparticles^8^, analysis of electrical circuit^9^. With time researchers presented many fractional derivative operators like Riemann–Liouville^10^, Caputo^11^, and Caputo–Fabrizio^12^, etc., but all these mentioned models were not applicable globally because of their local kernel. To fix the issue pointed out in the stated models, in 2016, Atangana and Baleanu^13^ introduced the Mittag–Leffler function to make the kernel of fractional derivative operator non-local. Al-kahtani^14^ used the newly suggested operator of fractional derivative to analyse the dynamics of Chua’s circuit law. Murtaza et al.^15^ inspected the exact solution of the non-linear Maxwell nanofluid flow. The authors associated their results in the field of concrete-based nanomaterials. They highlighted in their studies that fractional operator gives better description about the heredity of problem. Shuaib et al.^16^ addressed viscous fluid flow under the consequences of Soret and Dufour effect with energy transition due to fluctuating motion of an elastic rotating disc using the Caputo derivative. Li et al.^17^ addressed the fractional simulations for Darcy hybrid nanofluid flow across a perforated gyrating disc using the Matlab fractional algorithm Fde12. It has been concluded from the analysis that the efficiency of based fluid is remarkably enhances with the addition of nanoparticles. The hybrid nanofluid model of Brinkman-type fluid is studied by Shafie et al.^18^ by using the AB operator. Fractionalized Casson fluid-based Fourier’s and generalized Fick’s law has been examined by Sheikh et al.^19^. They used a new approach of fractional calculus in their calculation. In their study, they used the joint transformations of Fourier and Laplace transform for obtaining the exact solutions of the proposed model. Other important and significant studies regarding the implementations of fractional approach can be obtained in^20^.

In contrast to Newtonian fluids, non-Newtonian fluids depict the non-linear deformation rate in response of tangential stresses. The viscosity of the non-Newtonian fluids is change when shear stresses are acting on it. Examples of non-Newtonian fluids are ketchup, polymers, engine oil, transformer oil etc. Because of the immense uses in different areas of technology, the examination of non-Newtonian fluids has become an exciting topic. To explain the features of the flow of non-Newtonian fluids, the Navier Stoke’s equations are no more reliable. Therefore, researchers formulated various models like Maxwell model, Casson model, Jeffrey model, Brinkman-type fluid model etc. In the present research manuscript, we have been considered the Brinkman-type fluid model for the analysis of electro-osmotic flow of drilling nanofluids based on clay nanoparticles. Brinkman-type fluid model has vast number of applications in the area where permeability involved like petroleum reservoir, textile factories, grain storage, drilling liquids and heat pipes etc. Due to these mentioned applications Brinkman-type fluid model become very interesting to the mathematicians and researchers. The theoretical study and the corresponding mathematical model of the viscous fluid that is flowing in a highly saturated permeable medium were established by Darcy^21^. More precisely, the law elaborates the flow phenomenon that is flowing in the medium that contains saturated pores. Numerous studies have been done on the fluid flow problem in a porous medium using the Brinkman model. By making use of the Brinkman-type fluid model, the rheology of the fluid that is flowing in a porous channel has been presented in^22^. This problem was solved in two cases which are, (1) when both walls contain pores and (2) when the upper wall is stiff, and the lower wall is permeable. The flow through the channel is with high permeability and therefore Brinkman’s model has been considered. Von Karman's classic swirling flow over a permeable whirling disc with injection effect is adapted for Maxwell fluid by Zhou et al.^23^. The Maxwell nanofluid nature was described by Buongiorno's model, which combines both Brownian and thermophoresis motion. The mass propagation appears to increase exponentially as the thermophoresis component is elevated, but angular and radial velocities decline as the viscosity factor is enhanced. Theoretical and comparative analysis for two different kernels in the light of Brinkman-type fluid model has been done by Sarwar et al.^24^. The system of mathematical coupled PDEs has been developed by inserting constructive equations and then solved by perturbation techniques for two different fractional approaches. Saqib et al.^25^ discussed the shape effects of Fe_2_O_3_ on ferro nanofluids in the light of fractional Brinkman type fluid model. The authors also considered the ramped heating and heat generation influences in their inspection. The colloidal solution has been made by adding the Fe_2_O_3_ nanoparticles in water. The mathematical model for the considered phenomena has been developed in terms of linear coupled PDEs. Bilal et al.^26^ used an inverted extending cylinder to examine the Darcy convective flow of the CNTs and iron oxide hybrid nanofluid. The authors also assumed transverse magnetic field radiation of heat in their account. Local model of the considered phenomena has been artificially transformed to a non-local model by incorporating fractional approach. In their analysis the authors observed 6.35% efficiency in the Nusselt number against MoS_2_ nanoparticulate. The behavior of the Brinkman-type micropolar nanofluid motion in response of thermal radiation and nanoparticles is examined by Rafique et al.^27^. The authors obtained the solution of the assumed phenomena via numerical method i.e., Keller box technique. The authors highlighted in their study that Brinkman parameter reduces the velocity profile. Kumar et al.^28^ discussed the double diffusive hydromagnetic Brinkman type nanofluid flow under the influences of radiating energy and 1^st^ order chemical reaction. The authors considered ramped fashion and exponential accelerated plat. Numerical investigation of swirling flow and heat transfer of a nanofluid in a tube with helical ribs using a two-phase model has been done by Monfared et al.^28^. Numerical investigation of mixed convection of nanofluid flow in a trapezoidal channel with different aspect ratios in the presence of porous medium has been examined by Shorbagy et al.^29^ the features of stratification phenomena for 3D flow of Cross nanofluid considering activation energy has been analysed by Ali et al.^30^. Some relevant literature can be found in^31–35^.

Bearing in the mind of the above-stated literature survey, we have found that no one has been considered the Brinkman-type clay-based drilling nano liquid under the consequences of heat generation, electro-osmosis and chemical reaction. Therefore, to fill this gap we have assumed the electro-osmotic fractional model of Brinkman-type clay-based drilling nanofluid with the influence of transverse magnetic field and oscillating boundaries. The governing linear mathematical coupled partial differential equations are generalized by mean of fractional Caputo–Fabrizio derivative with the exponential kernel. The Laplace transforms are executed to determine the outcomes of the fundamental PDEs. Graphs and tables are deployed to present the findings.

### Physical description of the problem

**Figure 1:**
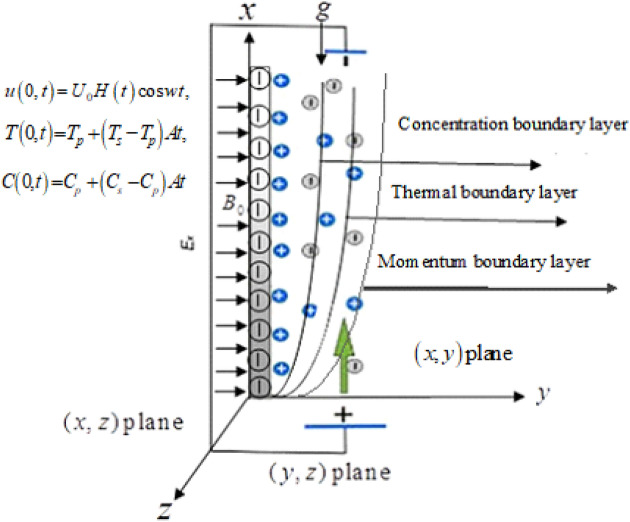
Flow Regime.

An incompressible time-dependent generalized electro-osmotic flow of Brinkman-type nanofluid has been considered on the vertical plate. The plate is taken along the *y*-axis which is normal to the *x*-axis. The fluid and plate are initially assumed to be stationary with the temperature $T_{s}$. At $t > 0$ in the *x*-direction, the fluid start motion and temperature rise up to $T_{p} + \left( {T_{p} - T_{s} } \right)At$, due to cosine oscillation of the plate. The Flow regime along with the appropriate initial and boundary conditions is shown in Fig. 1.

The governing equations that describe the flow phenomenon of Brinkman-type nanofluid are given by^36^.

1 $$ \begin{aligned} \rho_{nf} \left( {\frac{{\partial u\left( {y,t} \right)}}{\partial t} + \beta^{*} u\left( {y,t} \right)} \right) & = \mu_{nf} \frac{{\partial^{2} u\left( {y,t} \right)}}{{\partial y^{2} }} - \sigma_{nf} B_{0}^{2} u\left( {y,t} \right) + E_{x} \rho_{e} + g\left( {\rho \beta_{T} } \right)_{nf} \left( {T\left( {y,t} \right) - T_{s} } \right) \\ & \quad + g\left( {\rho \beta_{C} } \right)_{nf} \left( {C\left( {y,t} \right) - C_{s} } \right), \\ \end{aligned} $$

2 $$ \left( {\rho C_{p} } \right)_{nf} \frac{{\partial T\left( {y,t} \right)}}{\partial t} = k_{nf} \frac{{\partial^{2} T\left( {y,t} \right)}}{{\partial y^{2} }} + Q_{0} \left( {T - T_{s} } \right), $$

3 $$ \frac{\partial C}{{\partial t}} = D_{nf} \frac{{\partial^{2} C}}{{\partial y^{2} }} - k_{1} (C - C_{s} ), $$

subjected to the initial and boundary conditions:

4 $$ \left. {\begin{array}{*{20}l} {u\left( {y,0} \right) = 0,} \hfill & {T\left( {y,0} \right) = T_{s} ,} \hfill & {C\left( {y,0} \right) = C_{s} ,} \hfill \\ {u\left( {0,t} \right) = U_{0} H\left( t \right)\cos wt,} \hfill & {T\left( {0,t} \right) = T_{p} + \left( {T_{s} - T_{p} } \right)At,} \hfill & {C\left( {0,t} \right) = C_{p} + \left( {C_{s} - C_{p} } \right)At,} \hfill \\ {u\left( {\infty ,t} \right) = 0,} \hfill & {T\left( {\infty ,t} \right) = T_{s} .} \hfill & {C\left( {\infty ,t} \right) = C_{s} .} \hfill \\ \end{array} } \right\} $$

The governing equations consist of three mathematical equation that are Momentum equation, heat equation and concentration equation. Momentum equation is considered for the time dependent, free convection Brinkman-type nanofluid flow in the presence of external magnetic field, and electro-osmosis effect. The heat equation has been considered for heat flat plate in the presence of nanofluid effect and heat generation. The last term of the heat equation $Q_{0} \left( {T - T_{s} } \right)$ is for the effect of heat generation. While, Eq. (3) is taken for the effect of mass distribution on fluid flow. The concentration equation has been considered under the influence of chemical reaction. The term $k_{1} (C - C_{s} )$ represent the chemical reaction effect in the mass distribution equation.

Here $\rho_{e}$ shows the total charge density and *E*_*x*_ is external electric field. While, $\left( {\rho C_{p} } \right)_{nf}$ and $k_{nf}$ shows specific heat capacity and thermal conductivity of nanofluid respectively, while $Q_{0}$ is heat generation term and $k_{1}$ is chemical reaction parameter.

The term $\rho_{e}$ for infinite channel is defined as^15^:

5 $$ \rho_{e} = - \varepsilon k^{2} \psi_{p} e^{ - ky} ; $$

Here $\varepsilon$ is the dielectric permittivity of the solvent, $k^{2}$ represents the Debye–Huckel parameter and $\psi_{p}$ denotes zeta potential at the plate.

### Thermo-physical properties

This section concerns with the descriptions of the relation between nanoparticles and conventional base fluid.

### Dynamic viscosity

The mathematical relation between the dynamic viscosity $\mu_{nf}$ of the conventional base fluid and solid nano-size particles is given by Brinkman^37^.

6 $$ \mu_{nf} = \frac{{\mu_{f} }}{{\left( {1 - \phi } \right)^{2.5} }}. $$

### The effective density and mass diffusion rate

Based on Maxwell-Garnetts (MG) model, the density $\rho_{nf}$ of nanofluid is rebound as^38^:

7 $$ \rho_{nf} = \left( {1 - \phi } \right)\rho_{f} + \phi \rho_{s} .\quad D_{nf} = \left( {1 - \phi } \right)D_{f} $$

### The specific heat capacity and volumetric thermal and mass expansion

The mathematical relations for heat capacitance and the volumetric thermal expansion of conventional base fluid and nano-size particles are respectively given by Bourantas and Loukopoulos [32/39]:

8 $$ \left( {\rho C_{p} } \right)_{nf} = \left( {\rho C_{p} } \right)_{f} \left( {1 - \phi } \right) + \phi \left( {\rho C_{p} } \right)_{s} , $$

9 $$ \left( {\rho \beta_{T} } \right)_{nf} = \left( {\rho \beta_{T} } \right)_{f} \left( {1 - \phi } \right) + \phi \left( {\rho \beta_{T} } \right)_{s} .\quad \left( {\rho \beta_{C} } \right)_{nf} = \left( {\rho \beta_{C} } \right)_{f} \left( {1 - \phi } \right) + \phi \left( {\rho \beta_{C} } \right)_{s} . $$

### Electrical and thermal conductivity

The mathematical expressions for electrical and thermal conductivity of conventional nanofluid based on Maxwell’s model^40^ are given as:

10 $$ \sigma_{nf} = \sigma_{f} \left[ {1 + \frac{{3\left( {\frac{{\sigma_{s} }}{{\sigma_{f} }} - 1} \right)\phi }}{{\left( {\frac{{\sigma_{s} }}{{\sigma_{f} }} + 2} \right) - \left( {\frac{{\sigma_{s} }}{{\sigma_{f} }} - 1} \right)\phi }}} \right], $$

11 $$ k_{nf} = k_{f} \left[ {\frac{{k_{s} + 2k_{f} - 2\phi \left( {k_{f} - k_{s} } \right)}}{{k_{s} + 2k_{f} + 2\phi \left( {k_{f} - k_{s} } \right)}}} \right]. $$

### Generalization of local model

This section of the manuscript demonstrates the transformation of the local model into a generalized model. Primarily, the dimensional governing equations have been non-dimensionalized by using relative non-similarity variables to get rid of units and reduce variables. After that, the non-dimensional local model has been artificially transformed to a generalized model by utilizing the approach of time-fractional Caputo–Fabrizio derivative^10^. The fractional model is more convenient and general as compared to the local model for the elaboration of memory effect and flow behavior.

Introducing the following unit less similarity quantities:

12 $$ u^{*} = \frac{u}{{U_{0} }},\quad \xi = \frac{{U_{0} }}{\upsilon }y,\quad \tau = \frac{{U_{0}^{2} }}{\upsilon }t,\quad \Theta = \frac{{T - T_{S} }}{{T_{p} - T_{s} }},\quad \varphi = \frac{{C - C_{S} }}{{C_{p} - C_{s} }}. $$

Incorporating the above dimensionless quantities, Eqs. (1)–(4) takes the form;

13 $$ \frac{{\partial u\left( {\xi ,\tau } \right)}}{\partial \tau } + \beta u\left( {\xi ,\tau } \right) = b_{4} \frac{{\partial^{2} u\left( {\xi ,\tau } \right)}}{{\partial \xi^{2} }} - \lambda_{5} u\left( {\xi ,\tau } \right) + \lambda_{6} \Theta \left( {\xi ,\tau } \right) + \psi \varphi \left( {\xi ,\tau } \right) + Ese^{ - k\xi } , $$

14 $$ \frac{{\partial \Theta \left( {\xi ,\tau } \right)}}{\partial \tau } = \lambda_{4} \frac{{\partial^{2} \Theta \left( {\xi ,\tau } \right)}}{{\partial \xi^{2} }} + \chi \Theta \left( {\xi ,\tau } \right), $$

15 $$ \frac{{\partial \varphi \left( {\xi ,\tau } \right)}}{\partial \tau } = \frac{1}{Sc}\frac{{\partial^{2} \varphi \left( {\xi ,\tau } \right)}}{{\partial \xi^{2} }} - \alpha \varphi \left( {\xi ,\tau } \right), $$

along with:

16 $$ \left. {\begin{array}{*{20}l} {u\left( {\xi ,0} \right) = 0,} \hfill & {\Theta \left( {\xi ,0} \right) = 0,} \hfill & {\varphi \left( {\xi ,0} \right) = 0,} \hfill \\ {u\left( {0,\tau } \right) = H\left( \tau \right)\cos \omega \tau ,} \hfill & {\Theta \left( {0,\tau } \right) = \tau ,} \hfill & {\varphi \left( {0,\tau } \right) = \tau ,} \hfill \\ {u\left( {\infty ,\tau } \right) = 0,} \hfill & {\Theta \left( {\infty ,\tau } \right) = 0,} \hfill & {\varphi \left( {\infty ,\tau } \right) = 0.} \hfill \\ \end{array} } \right\}. $$

where

$$ \begin{aligned} \beta & = \frac{{\beta^{*} \upsilon_{f} }}{{U_{0}^{2} }},\,\,\,\,\,\,\,M = \frac{{\sigma_{f} B_{0}^{2} \upsilon_{f} }}{{\rho_{f} U_{0}^{2} }},\,\,\,\,\,\,\,Gr = \frac{{\upsilon_{f} (\beta_{T} )_{f} g\left( {T_{P} - T_{s} } \right)}}{{U_{0}^{3} }},\,\,\,\,\,\,\,\,Gm = \frac{{\upsilon_{f} (\beta_{C} )_{f} g\left( {C_{P} - C_{s} } \right)}}{{U_{0}^{3} }},\,\,\,\,\,\,\, \\ Es & = \frac{{\upsilon_{f} E_{x} \varepsilon k^{2} \psi_{p} }}{{U_{0}^{3} \rho_{f} }},\,\,\,\Pr = \frac{{\mu_{f} (C_{p} )_{f} }}{{k_{f} }},\,\,\,\,\,\chi = \frac{{Q_{0} \upsilon_{f} }}{{\left( {\rho C_{p} } \right)_{{_{f} }} \lambda_{1} U_{0}^{2} }},\,\,\,\,\,\alpha = \frac{{k_{1} \upsilon_{f} }}{{U_{0}^{2} }},\,\,\,\,\,\,Sc = \frac{{\nu_{f} }}{{\left( {1 - \phi } \right)D_{f} }},\,\,\, \\ b_{0} & = \left( {1 - \phi } \right) + \phi \frac{{\rho_{s} }}{{\rho_{f} }},\,\,\,\,\,b_{1} = \mu_{f} \left( {1 - \phi } \right)^{ - 2.5} ,\,\,b_{2} = \left( {1 - \phi } \right) + \phi \frac{{\left( {\rho \beta_{T} } \right)_{s} }}{{\left( {\rho \beta_{T} } \right)_{f} }},\,\,\,\,\,b_{3} = 1 + \frac{{3\left( {\frac{{\sigma_{s} }}{{\sigma_{f} }} - 1} \right)\phi }}{{\left( {\frac{{\sigma_{s} }}{{\sigma_{f} }} + 2} \right) - \left( {\frac{{\sigma_{s} }}{{\sigma_{f} }} - 1} \right)\phi }},\, \\ b_{4} & = \frac{{b_{1} }}{{b_{0} }},\,\,\,\,\,\,\,\,b_{5} = \frac{{b_{3} }}{{b_{0} }},\,\,\,\,\,\,\,b_{6} = \frac{{b_{2} }}{{b_{0} }},\,\,\,\,\,\lambda_{1} = \left( {1 - \phi } \right) + \phi \frac{{\left( {\rho C_{p} } \right)_{s} }}{{\left( {\rho C_{p} } \right)_{f} }},\,\,\,\,\,\,\lambda_{2} = \frac{{k_{s} + 2k_{f} - 2\phi \left( {k_{f} - k_{s} } \right)}}{{k_{s} + 2k_{f} + 2\phi \left( {k_{f} - k_{s} } \right)}},\,\,\,\,\,\,\, \\ \lambda_{3} & = \frac{{\lambda_{2} }}{{\lambda_{1} }},\,\,\,\,\,\,\,\,\lambda_{4} = \frac{{\lambda_{3} }}{\Pr },\,\,\,\,\,\,\,\,\lambda_{5} = Mb_{5} ,\,\,\,\lambda_{6} = b_{6} Gr.\,\,\,\,\psi = Gm\,\alpha_{0} ,\,\,\,\,\,\,\,\,\alpha_{0} = \frac{{\alpha^{*} }}{{b_{0} }},\,\,\,\,\,\alpha^{*} = \left( {1 - \phi } \right) + \phi \frac{{\left( {\rho \beta_{C} } \right)_{s} }}{{\left( {\rho \beta_{C} } \right)_{f} }}, \\ \end{aligned} $$

are the Brinkman parameter, magnetic (Resistive) parameter, thermal Grashoff number, mass Grashoff number, electro-osmotic parameter, Prandtl number, heat generation parameter, chemical reaction parameter and Schmidth number respectively, while $b_{0} ,\,\,b_{1} ,\,\,b_{2} ,\,\,b_{3} ,\,\,b_{4} ,\,\,b_{5} ,\,\,b_{6} ,\,\,\lambda_{1} ,\,\,\lambda_{2} ,\,\,\lambda_{3} ,\,\lambda_{4} ,\lambda_{5} \;{\text{and}}\;\lambda_{6}$ are the constants produced during calculation. The time-fractional Caputo–Fabrizio model of the unit less governing Eqs. (13), (14) and (15) is given as;

17 $$^{CF} \wp_{\tau }^{\gamma } u\left( {\xi ,\tau } \right) + \beta u\left( {\xi ,\tau } \right) = b_{4} \frac{{\partial^{2} u\left( {\xi ,\tau } \right)}}{{\partial \xi^{2} }} - \lambda_{5} u\left( {\xi ,\tau } \right) + \lambda_{6} \Theta \left( {\xi ,\tau } \right) + \psi \varphi \left( {\xi ,\tau } \right) - Ese^{ - k\xi } , $$

18 $$^{CF} \wp_{\tau }^{\gamma } \Theta \left( {\xi ,\tau } \right) = \lambda_{4} \frac{{\partial^{2} \Theta \left( {\xi ,\tau } \right)}}{{\partial \xi^{2} }} + \chi \Theta \left( {\xi ,\tau } \right). $$

19 $$^{CF} \wp_{\tau }^{\gamma } \varphi \left( {\xi ,\tau } \right) = \frac{1}{Sc}\frac{{\partial^{2} \varphi \left( {\xi ,\tau } \right)}}{{\partial \xi^{2} }} - \alpha \varphi \left( {\xi ,\tau } \right), $$

In Eqs. (17), (18) and (19) $^{CF} \wp_{\tau }^{\gamma }$ is the time-fractional Caputo–Fabrizio operator and defined as^10^;

20 $$^{CF} \wp_{\tau }^{\gamma } g\left( {t^{ * } } \right) = \frac{M\left( \gamma \right)}{{1 - \gamma }}\int\limits_{0}^{{t^{ * } }} {\exp \left( { - \frac{{\gamma \left( {t^{*} - \tau } \right)}}{1 - \gamma }} \right)g^{ \cdot } \left( {t^{*} } \right)} d\tau ,\quad 0 < \gamma < 1. $$

In the present study, two very important properties of the CF operator will be used.

1. $M\left( \gamma \right)$ is the normalization function^43^:
  21 $$ M\left( 1 \right) = M\left( 0 \right) = 1. $$
2. The Laplace transform property of the Eq. (20) will be utilized such that;
  22 $$ \pounds\left\{ {^{CF} \wp_{\tau }^{\gamma } g\left( \tau \right)} \right\}\left( s \right) = \frac{{s\overline{g}\left( s \right) - g\left( 0 \right)}}{{\left( {1 - \gamma } \right)s + \gamma }},\quad 0 < \gamma < 1, $$
  where $\overline{g}\left( s \right)$ is the Laplace transform of the function $g\left( \tau \right)$.

### Solution of the energy equation

Utilizing the the Laplace transform property addressed in Eq. (22) initial condition given in Eq. (16) Eq. (18) will take the shape of;

23 $$ \frac{{d^{2} \Theta \left( {\xi ,\tau } \right)}}{{d\xi^{2} }} - \left( {\frac{{\delta_{2} q - a_{4} }}{{q + \delta_{1} }}} \right) = 0, $$

the transformed conditions are:

24 $$ \left. {\overline{\Theta } \left( {0,q} \right) = \frac{1}{q},\,\,\,\,\,\,\,\,\,\,\overline{\Theta } \left( {\infty ,q} \right) = 0} \right\}. $$

Using boundary conditions written in Eq. (24), the exact solution of the Eq. (23) is given by:

25 $$ \overline{\Theta } \left( {\xi ,q} \right) = \frac{1}{q}\exp \left( { - \xi \sqrt {\frac{{\delta_{2} q - a_{4} }}{{q + \delta_{1} }}} } \right), $$

with

$$ a_{0} = \left( {1 - \gamma } \right)^{ - 1} ,\,\,\,\,\,\,\,\,\delta_{1} = a_{0} \gamma ,\,\,\,\,\,\,\,\,\delta_{2} = \frac{{a_{0} }}{{\lambda_{4} }},\,\,\,\,\,\,\,a_{2} = a_{0} - \chi ,\,\,\,\,\,\,a_{3} = a_{1} \chi ,\,\,\,\,\,a_{4} = \frac{{a_{3} }}{{\lambda_{4} }}. $$

Equation (25) can be simply expressed as:

26 $$ \overline{\Theta } \left( {\xi ,q} \right) = \overline{{\chi_{1} }} \left( {\xi ,q,0,\delta_{2} , - a_{4} ,\delta_{1} } \right), $$

where

27 $$ \overline{{\chi_{1} }} \left( {\xi ,q,s_{1} ,s_{2} ,s_{3} ,s_{4} } \right) = \frac{1}{{q + s_{1} }}\exp \left( { - \xi \left( {\sqrt {\frac{{qs_{2} + s_{3} }}{{q + s_{4} }}} } \right)} \right), $$

Equation (26) is the exact solution of the fractional order energy equation in the domain of Laplace transform. In order to attain the exact solution in time domain, we will apply the inverse Laplace transform on the Eq. (26), we get the final exact solution in the form:

28 $$ \Theta \left( {\xi ,\tau } \right) = \chi_{1} \left( {\xi ,\tau ,0,\delta_{2} , - a_{4} ,\delta_{1} } \right), $$

here

29 $$ \chi_{1} \left( {\xi ,t,s_{1} ,s_{2} ,s_{3} ,s_{4} } \right) = {\text{e}}^{{ - lt - \sqrt {s_{2} } }} - \frac{{z\sqrt {s_{3} - s_{2} s_{4} } }}{2\sqrt \pi }\int\limits_{0}^{t} {\int\limits_{0}^{\infty } \begin{gathered} \frac{{{\text{e}}^{{ - s_{1} t}} }}{\sqrt \tau }\exp \left( {s_{1} \tau - s_{4} \tau - \frac{{z^{2} }}{4u} - s_{2} u} \right) \hfill \\ I_{1} \left( {2\sqrt {\left( {s_{3} - s_{2} s_{4} } \right)u\tau } } \right)d\tau du. \hfill \\ \end{gathered} } . $$

### Solution of the concentration equation

Utilizing the the Laplace transform property addressed in Eq. (22) and initial condition given in Eqs. (16), (19) will take the shape of;

30 $$ \frac{{d^{2} \overline{\varphi } \left( {\xi ,q} \right)}}{{d\xi^{2} }} - \left( {\frac{{\delta_{2} q + \eta }}{{\delta_{1} + q}}} \right)\overline{\varphi } \left( {\xi ,q} \right) = 0. $$

the transformed conditions are:

31 $$ \left. {\overline{\varphi } \left( {0,q} \right) = \frac{1}{q},\,\,\,\,\,\,\,\,\,\,\overline{\varphi } \left( {\infty ,q} \right) = 0} \right\}. $$

Using boundary conditions written in Eq. (31), the exact solution of the Eq. (30) is given by:

32 $$ \overline{\varphi } \left( {\xi ,q} \right) = \frac{1}{q}\exp \left( { - \xi \sqrt {\frac{{\delta_{2} q + \eta }}{{q + \delta_{1} }}} } \right), $$

Equation (32) can be simply written as:

33 $$ \overline{\varphi } \left( {\xi ,q} \right) = \overline{{\chi_{1} }} \left( {\xi ,q,0,\delta_{2} ,\eta ,\delta_{1} } \right), $$

Equation (23) is the exact solution of the fractional order energy equation in the domain of Laplace transform. In order to attain the exact solution in time domain, we will apply the inverse Laplace transform on the Eq. (23), we get the final exact solution in the form:

34 $$ \varphi \left( {\xi ,\tau } \right) = \chi_{1} \left( {\xi ,\tau ,0,\delta_{2} ,\eta ,\delta_{1} } \right), $$

### Solution of the momentum equation

To obtain the exact solution of the unit less time-fractional momentum equation, the Laplace transform is applied on Eq. (17) along with relative boundary conditions, we arrived at:

35 $$ \frac{{d^{2} \overline{u} \left( {\xi ,q} \right)}}{{d\xi^{2} }} - \left( {\frac{{d_{0} q + d_{1} }}{{q + \delta_{1} }}} \right)\overline{u} \left( {\xi ,q} \right) = - d_{2} \overline{\Theta } \left( {\xi ,q} \right) - \psi_{1} \overline{\varphi } \left( {\xi ,q} \right) + \frac{{Es^{*} e^{ - k\xi } }}{q}, $$

36 $$ \left. {\overline{u} \left( {0,q} \right) = \frac{q}{{q^{2} + \omega^{2} }},\,\,\,\,\,\,\,\,\,\,\,\,\overline{u} \left( {\infty ,q} \right) = 0} \right\}. $$

Utilizing the transformed boundary conditions given in (36), the exact solution of Eq. (35) is given as:

37 $$ \begin{aligned} \overline{u} \left( {\xi ,q} \right) & = \left[ {\frac{q}{{q^{2} + \omega^{2} }} + \frac{{d_{2} \left( {q + \delta_{1} } \right)}}{{q\left( {d_{3} q - d_{1} } \right)}} + \frac{{\psi_{1} \left( {q + \delta_{1} } \right)}}{{q\left( {d_{3} q - d_{1} } \right)}} - \frac{{Es\left( {q + \delta_{1} } \right)}}{{q\left( {d_{4} q + d_{5} } \right)}}} \right]\exp \left( { - \xi \sqrt {\frac{{d_{0} q + d_{1} }}{{q + \delta_{1} }}} } \right) \\ & \quad - \frac{{d_{2} \left( {q + \delta_{1} } \right)}}{{q\left( {d_{3} q - d_{1} } \right)}}\exp \left( { - \xi \sqrt {\frac{{\delta_{2} q - a_{4} }}{{q + \delta_{1} }}} } \right) - \frac{{\psi_{1} \left( {q + \delta_{1} } \right)}}{{q\left( {d_{3} q - d_{1} } \right)}}\exp \left( { - \xi \sqrt {\frac{{\delta_{2} q + \eta }}{{q + \delta_{1} }}} } \right) \\ & \quad + \frac{{Es\left( {q + \delta_{1} } \right)}}{{q\left( {d_{4} q + d_{5} } \right)}}e^{ - k\xi } , \\ \end{aligned} $$

After using Partial fraction, Eq. (37) takes the form:

38 $$ \begin{aligned} \overline{u} \left( {\xi ,q} \right) & = \frac{q}{{q^{2} + \omega^{2} }}\exp \left( { - \xi \sqrt {\frac{{d_{0} q + d_{1} }}{{q + \delta_{1} }}} } \right) + \frac{{d_{8} }}{q}\exp \left( { - \xi \sqrt {\frac{{d_{0} q + d_{1} }}{{q + \delta_{1} }}} } \right) + \frac{{\psi_{1} }}{{d_{3} q - d_{1} }}\exp \left( { - \xi \sqrt {\frac{{d_{0} q + d_{1} }}{{q + \delta_{1} }}} } \right) \\ & \quad + \frac{{d_{9} }}{{d_{3} q - d_{1} }}\exp \left( { - \xi \sqrt {\frac{{d_{0} q + d_{1} }}{{q + \delta_{1} }}} } \right) - \frac{{l_{2} }}{q}\exp \left( { - \xi \sqrt {\frac{{d_{0} q + d_{1} }}{{q + \delta_{1} }}} } \right) - \frac{{l_{3} }}{{d_{4} q - d_{5} }}\exp \left( { - \xi \sqrt {\frac{{d_{0} q + d_{1} }}{{q + \delta_{1} }}} } \right) \\ & \quad - \frac{{d_{8} }}{q}\exp \left( { - \xi \sqrt {\frac{{\delta_{2} q - a_{4} }}{{q + \delta_{1} }}} } \right) - \frac{{d_{9} }}{{d_{3} q - d_{1} }}\exp \left( { - \xi \sqrt {\frac{{\delta_{2} q - a_{4} }}{{q + \delta_{1} }}} } \right) - \frac{{\psi_{1} }}{{d_{3} q - d_{1} }}\exp \left( { - \xi \sqrt {\frac{{\delta_{2} q + \eta }}{{q + \delta_{1} }}} } \right) \\ & \quad + \frac{{l_{2} }}{q}e^{ - k\xi } + \frac{{l_{3} }}{{d_{4} q - d_{5} }}e^{ - k\xi } \\ \end{aligned} $$

In more convenient form Eq. (38) can be expressed as:

39 $$ \begin{aligned} \overline{u} \left( {\xi ,q} \right) & = \frac{q}{{q^{2} + \omega^{2} }}\overline{{\chi_{1} }} \left( {\xi ,q,0,d_{0} ,d_{1} ,\delta_{1} } \right) + d_{8} \overline{{\chi_{1} }} \left( {\xi ,q,0,d_{0} ,d_{1} ,\delta_{1} } \right) + \psi_{1} \overline{{\chi_{1} }} \left( {\xi ,q,0,d_{0} ,d_{1} ,\delta_{1} } \right) \\ & \quad + l_{6} \overline{{\chi_{1} }} \left( {\xi ,q, - l_{7} ,d_{0} ,d_{1} ,\delta_{1} } \right) - l_{2} \overline{{\chi_{1} }} \left( {\xi ,q,0,d_{0} ,d_{1} ,\delta_{1} } \right) - l_{4} \overline{{\chi_{1} }} \left( {\xi ,q,l_{5} ,d_{0} ,d_{1} ,\delta_{1} } \right) \\ & \quad - d_{8} \overline{{\chi_{1} }} \left( {\xi ,q,0,\delta_{2} , - a_{4} ,\delta_{1} } \right) - l_{6} \overline{{\chi_{1} }} \left( {\xi ,q, - l_{7} ,\delta_{2} ,0,\delta_{1} } \right) + l_{2} R_{(\alpha ,0)} ( - 0,q)e^{ - \xi y} \\ & \quad + l_{3} R_{(\alpha ,0)} ( - l_{5} ,q)e^{ - \xi y} \\ \end{aligned} $$

As Eq. (38) is the exact solution of time-fractional momentum equation in Laplace transform domain, so to obtain the exact solution in the time domain, we apply the inverse Laplace transform on Eq. (39), we arrived at:

40 $$ \begin{aligned} u\left( {\xi ,\tau } \right) & = \cos \omega \tau *\chi_{1} \left( {\xi ,\tau ,0,d_{0} ,d_{1} ,\delta_{1} } \right) + d_{8} \chi_{1} \left( {\xi ,\tau ,0,d_{0} ,d_{1} ,\delta_{1} } \right) + \psi_{1} \chi_{1} \left( {\xi ,\tau ,0,d_{0} ,d_{1} ,\delta_{1} } \right) \\ & \quad + l_{6} \chi_{1} \left( {\xi ,\tau , - l_{7} ,d_{0} ,d_{1} ,\delta_{1} } \right) - l_{2} \chi_{1} \left( {\xi ,\tau ,0,d_{0} ,d_{1} ,\delta_{1} } \right) - l_{4} \chi_{1} \left( {\xi ,\tau ,l_{5} ,d_{0} ,d_{1} ,\delta_{1} } \right) \\ & \quad - d_{8} \chi_{1} \left( {\xi ,\tau ,0,\delta_{2} , - a_{4} ,\delta_{1} } \right) - l_{6} \chi_{1} \left( {\xi ,\tau , - l_{7} ,\delta_{2} ,0,\delta_{1} } \right) + l_{2} R_{(\alpha ,0)} ( - 0,\tau )e^{ - \xi y} \\ & \quad + l_{3} R_{(\alpha ,0)} ( - l_{5} ,\tau )e^{ - \xi y} . \\ \end{aligned} $$

With

$$\begin{aligned} \lambda_{7} & = \beta + \lambda_{5} ,\,\,\,\,\,\,\lambda_{8} = \lambda_{7} \delta_{1} ,\,\,\,\,\,\,\lambda_{9} = \delta_{0} + \lambda_{7} ,\,\,\,\,\,\,Es^{*} = \frac{Es}{{b_{4} }},\,\,\,\,\,\,d_{0} = \frac{{\lambda_{9} }}{{b_{4} }},\,\,\,\,\,\,d_{1} = \frac{{\lambda_{8} }}{{b_{4} }},\,\,\,\,\,\,d_{2} = \frac{{\lambda_{6} }}{{b_{4} }}, \\ d_{3} & = \delta_{2} - d_{0} ,\,\,\,d_{4} = k^{2} - d_{0} ,\,\,\,\,\,\,d_{5} = k^{2} \delta_{1} - d_{1} ,\,\,\,\,\,\,d_{6} = \frac{{ - \delta_{1} }}{{d_{1} }},\,\,\,\,\,\,d_{7} = \frac{{d_{1} + d_{3} \delta_{1} }}{{d_{1} }}, \\ d_{8} & = d_{2} d_{6} ,d_{9} = d_{2} d_{7} ,\,\,\,\,\,\,l_{0} = \frac{{\delta_{1} }}{{d_{5} }},\,\,\,\,\,l_{1} = \frac{{d_{5} - d_{4} \delta_{1} }}{{d_{5} }},\,\,\,\,\,\,l_{2} = Es^{*} l_{0} ,\,\,\,\,\,\,l_{3} = Es^{*} l_{1} ,\,\,\,\,\,l_{4} = \frac{{l_{3} }}{{d_{4} }}, \\ l_{5} & = \frac{{d_{4} }}{{d_{5} }},\,\,\,\,\,l_{6} = \frac{{d_{9} }}{{d_{3} }},\,\,\,\,\,\,l_{7} = \frac{{d_{1} }}{{d_{3} }}. \\ \end{aligned}$$

### Nusselt number

In unit less form the rate of heat transfer (Nusselt number) is given by;

41 $$ {\text{Nu}} = \lambda_{2} \left. {\frac{\partial \Theta }{{\partial \xi }}} \right|_{\xi = 0} , $$

### Sherwood number

In unit less form the rate of mass transfer (Sherwood number) is represented as:

42 $$ {\text{Sh}} = D_{nf} \left. {\frac{\partial \psi }{{\partial \xi }}} \right|_{\xi = 0} $$

## Results and discussion

The impact of introduced factors on velocity, energy, and mass propagation profile is discussed in this section. The classical governing equations have been altered to a fractional-order model by the mean of the Caputo–Fabrizio operator. After transformation, the exact solutions have been established through the technique of Laplace transformation.

**Figure 2:**
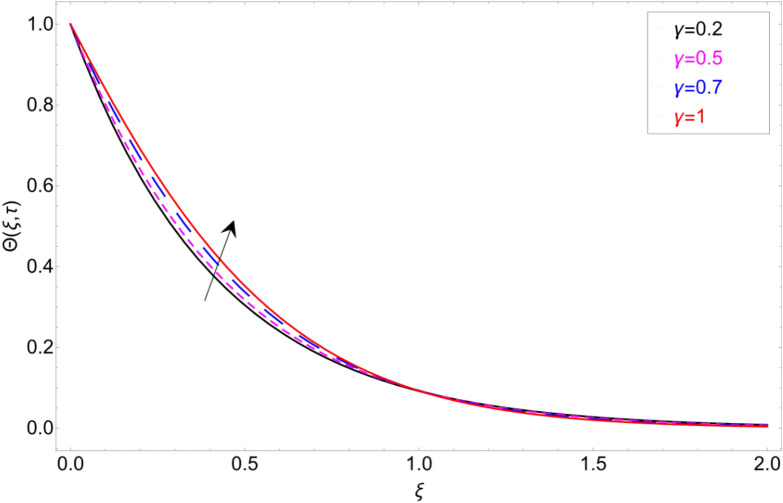
Temperature profile against fractional parameter $\gamma$.

**Figure 3:**
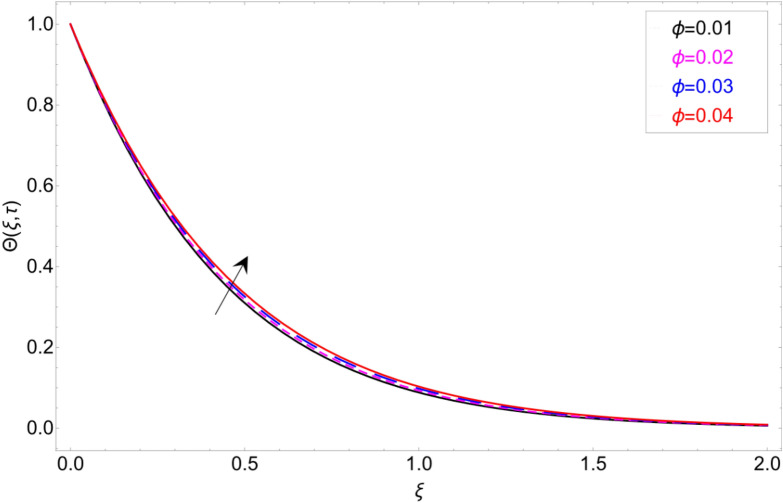
Temperature profile against volume fraction $\varphi$.

**Figure 4:**
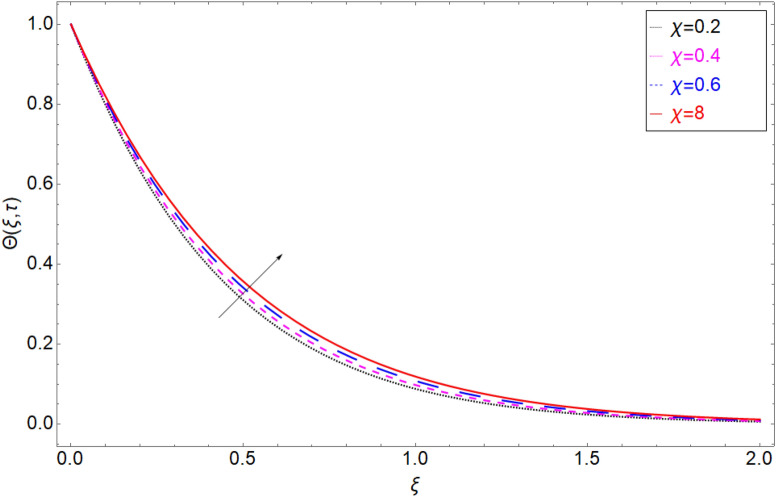
Temperature profile against Heat Generation parameter $\chi$.

**Figure 5:**
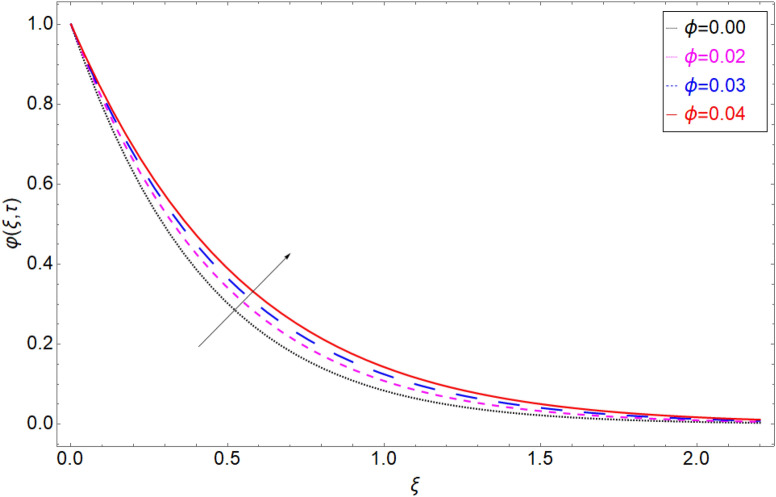
Mass transition profile versus the volume fraction coefficient $\phi$.

**Figure 6:**
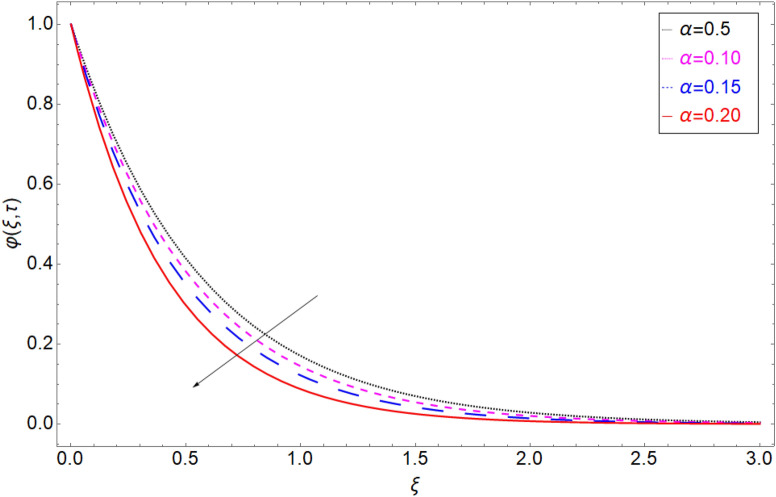
Mass transition profile versus the chemical reaction term $\alpha$.

**Figure 7:**
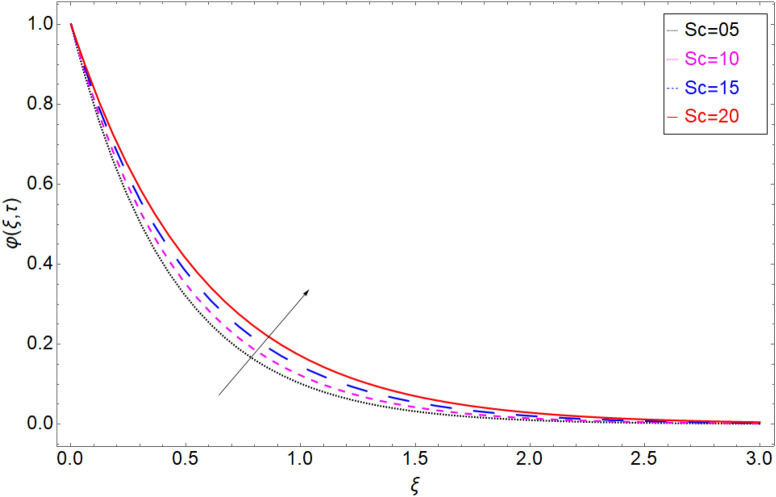
Mass transition profile versus the chemical reaction term $Sc$.

The behaviour of the temperature profile in response to the fractional parameter $\gamma$ is shown in Fig. 2. In fractional order derivative, we can draw more than one profile on different values of $\gamma$, and due to this prime advantage, the non-integer order derivative can provide different layers for investigation of the fluid. More generally for the fractional model, we fixed all the physical parameter fixed and check the variation of fractional parameter which shows the memory effect generally which relate the mathematical work with experimental work more accurate. The response of temperature profile against volume fraction $\phi$ of nanoparticles is plotted in Fig. 3. It highlighted the increasing behaviour on the temperature. As a result, the denser thermal boundary layer, which grows in proportion to the volume fraction of nanomaterials. Figure 4 has been sketched to check variation in thermal field against heat generation parameter $\chi$. Increase in thermal field has been reported against greater magnitude of $\chi$. As the value of $\chi$ increases the thermal conduction property of the fluid increases due to which the profile of heat transfer increase. Figure 5 shows variation in concentration field against volume fraction $\phi$. From the sketch, enhancement in the profile of mass concentration has been reported when the values of $\phi$ gradually increase from 0 to 0.04. This is physically true because greater volume fraction makes the fluid denser which consequently increase the concentration rate in the fluid and hence rise in concentration field has been observed. The same trend has also been observed in Fig. 6 for larger values of chemical reaction parameter $\alpha$. The effect of *Sc* has been portrayed in Fig. 7. Larger values of *Sc* show enlargement in the concentration profile and this is due to increase in the viscous forces and density in the fluid which makes the concentration rate boost up.

**Figure 8:**
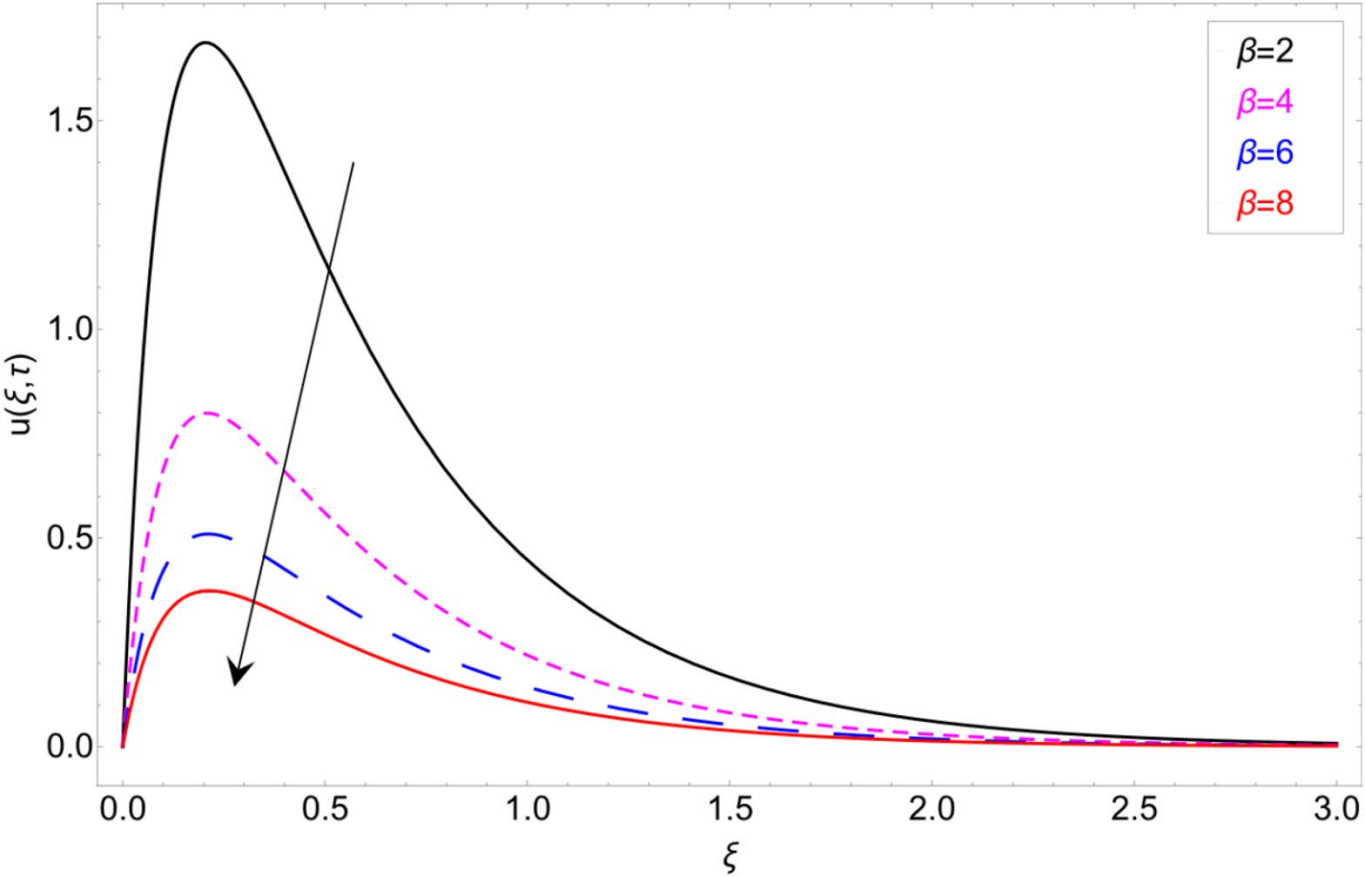
Velocity profile against Brinkman parameter $\beta$.

**Figure 9:**
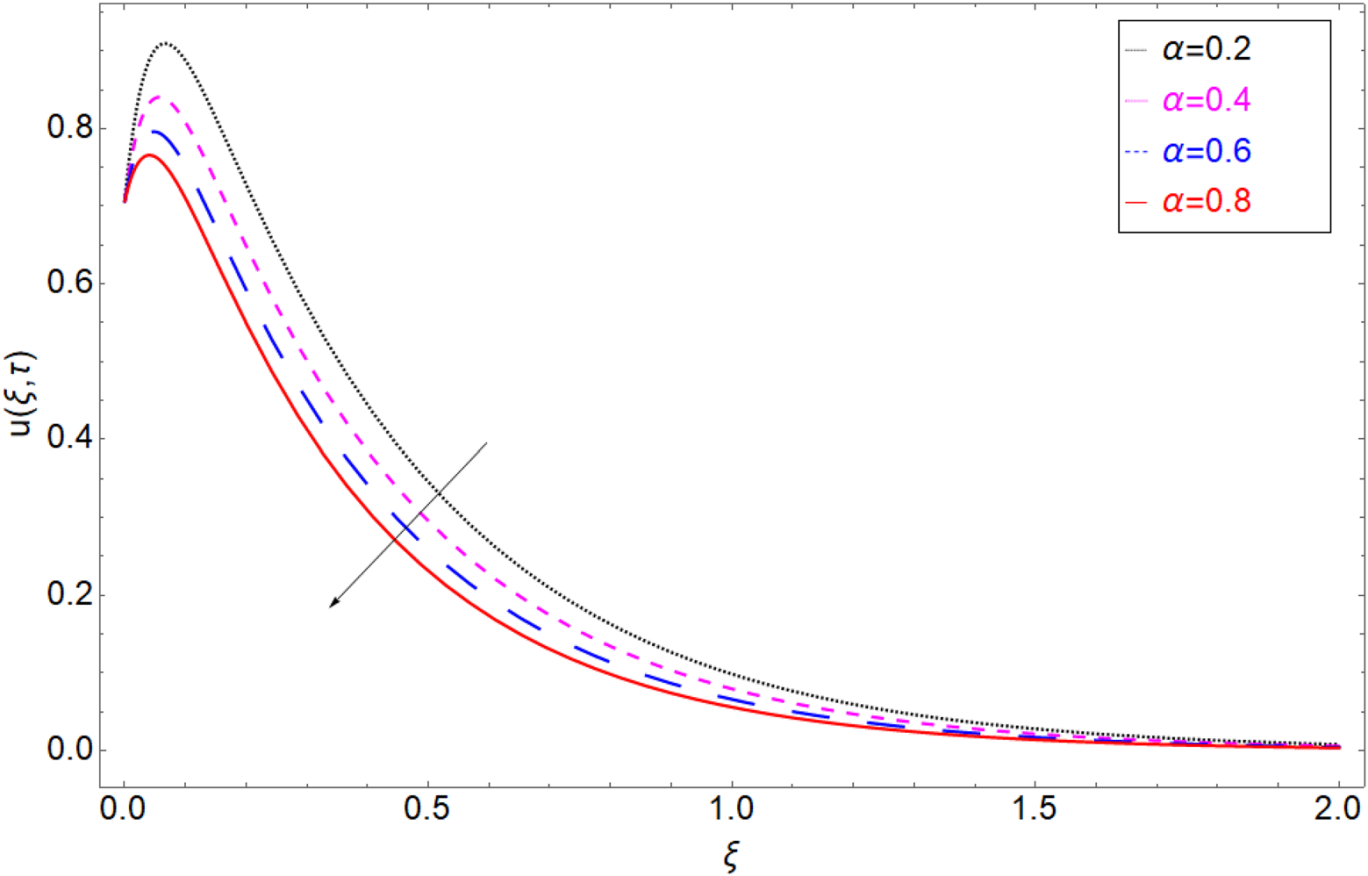
Velocity profile against chemical reaction parameter $\alpha$.

**Figure 10:**
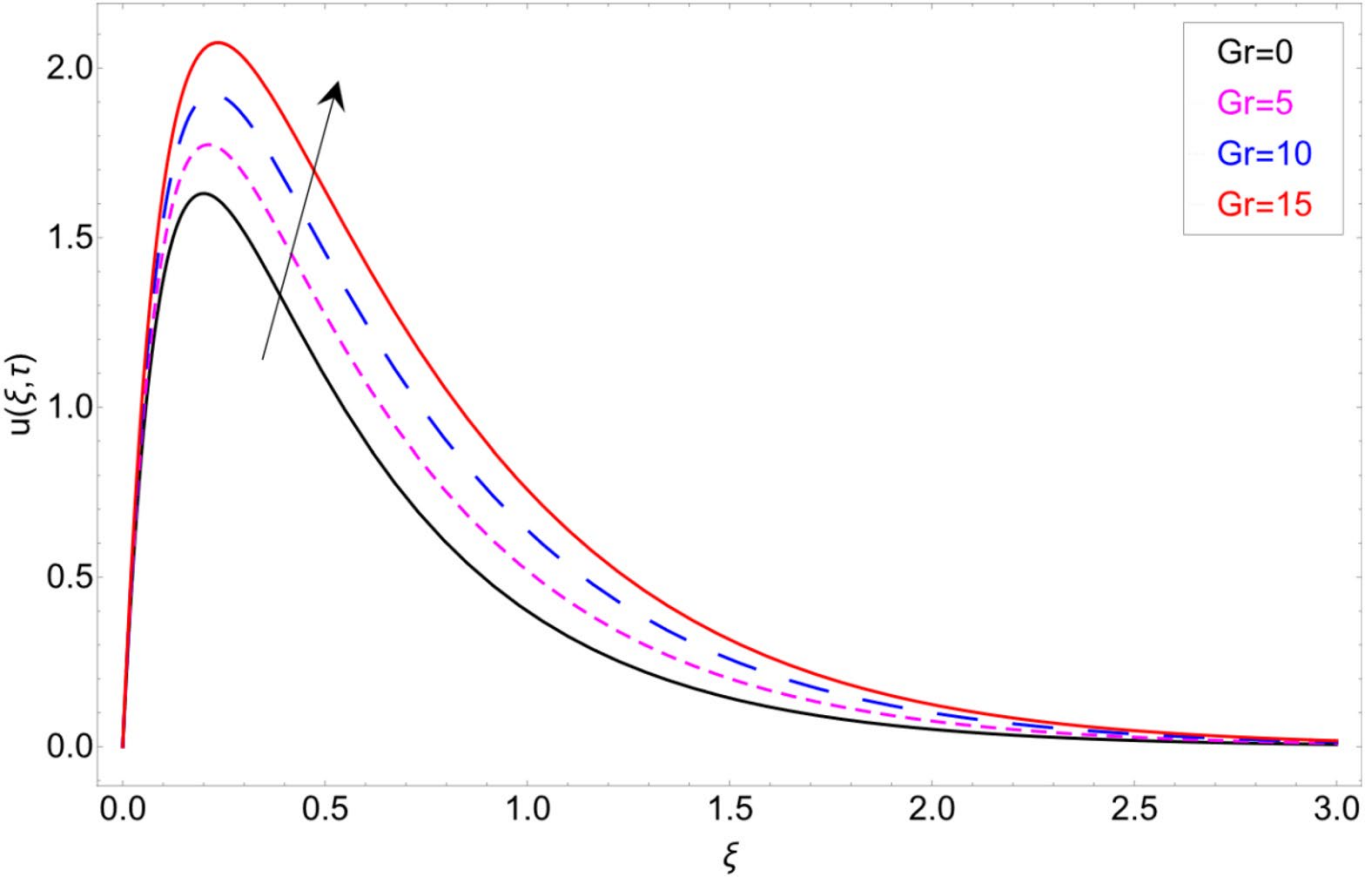
Velocity profile against thermal Grashof number *Gr.*

**Figure 11:**
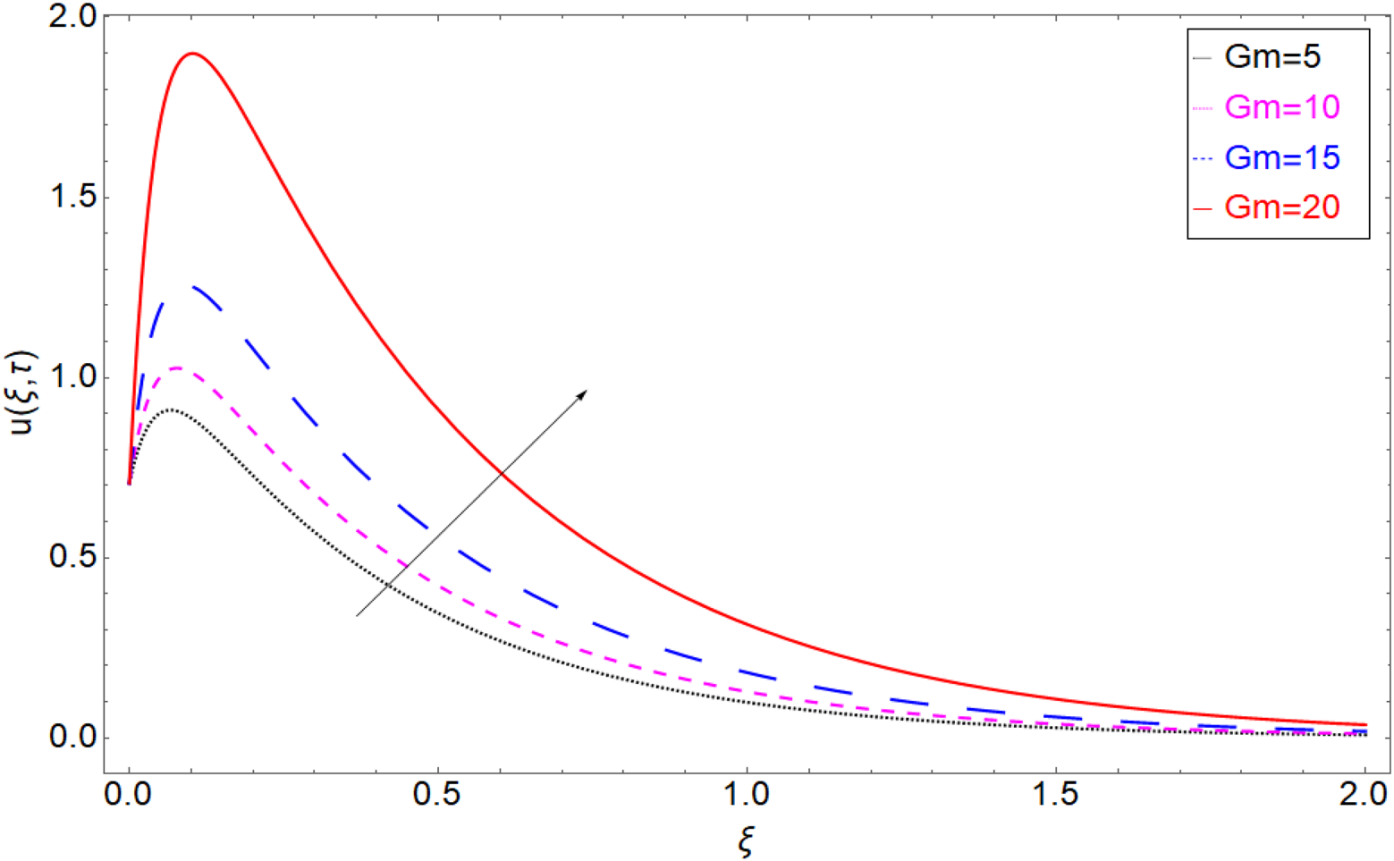
Velocity profile against mass Grashof number *Gm.*

To check the behaviour of velocity profile against Brinkman parameter $\beta$ Fig. 8 has been plotted. The Brinkman parameter represents the relation between the drag forces and the density of the fluid. As the magnitude of the Brinkman parameter increases the drag forces in the fluid increase which consequently retards the fluid motion and declination in the velocity profile is observed. Figure 9 represents declination in velocity profile against larger values of chemical reaction parameter $\alpha$. Physically it is true because greater magnitude of $\alpha$ makes the fluid denser and more viscous which consequently decrease the fluid motion. Figure 10 is portrayed to observe the velocity profile in the response of the thermal Grashof number. From the figure, it is perceived that the higher values of *Gr* show an increasing trend. This is because of buoyancy forces occurring in the fluid which lead to accelerating the fluid motion. The same behaviour also has been examined for mass Grahsof number in Fig. 11.

**Figure 12:**
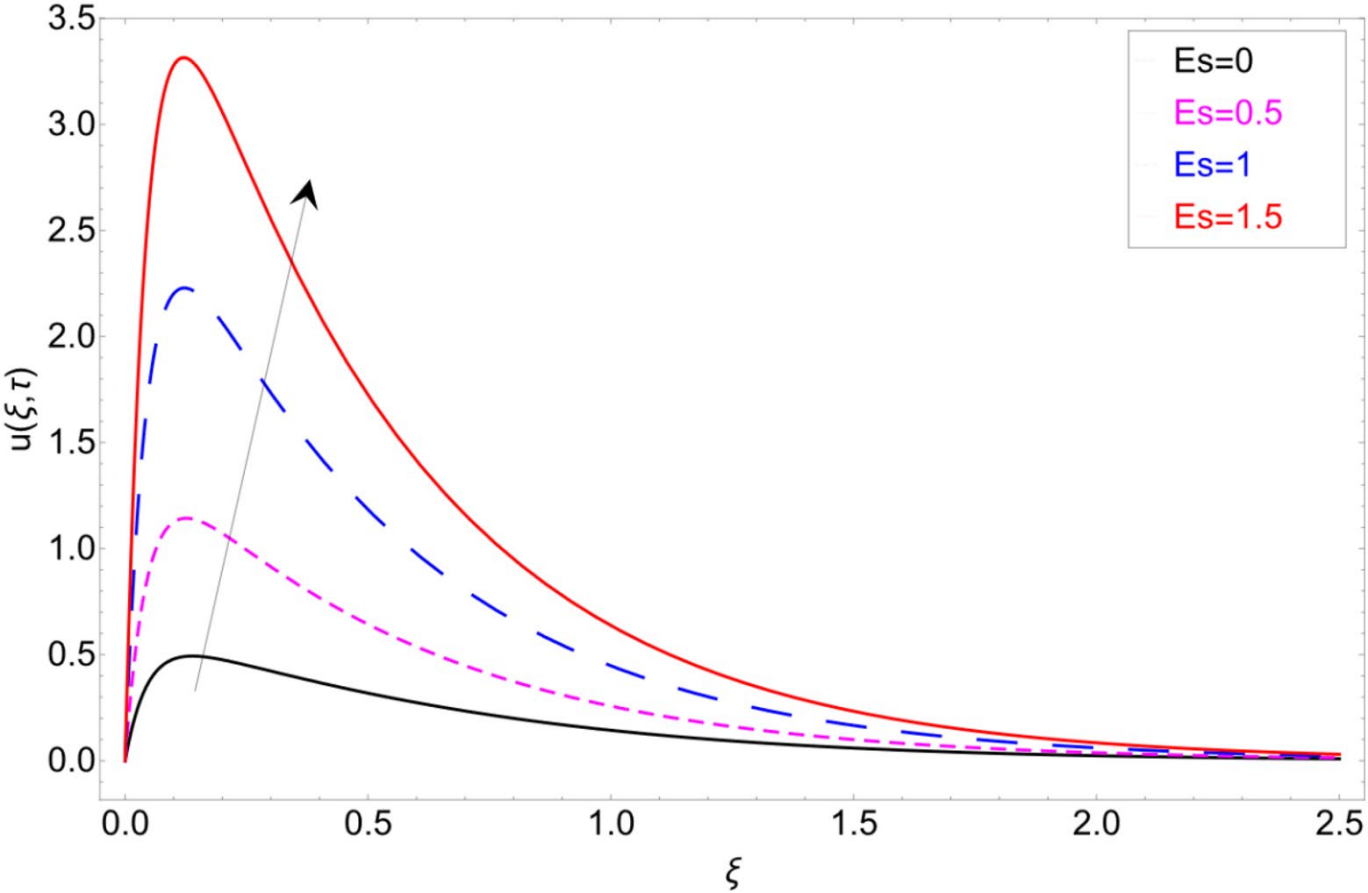
Velocity profile against Electro-osmotic parameter *Es.*

**Figure 13:**
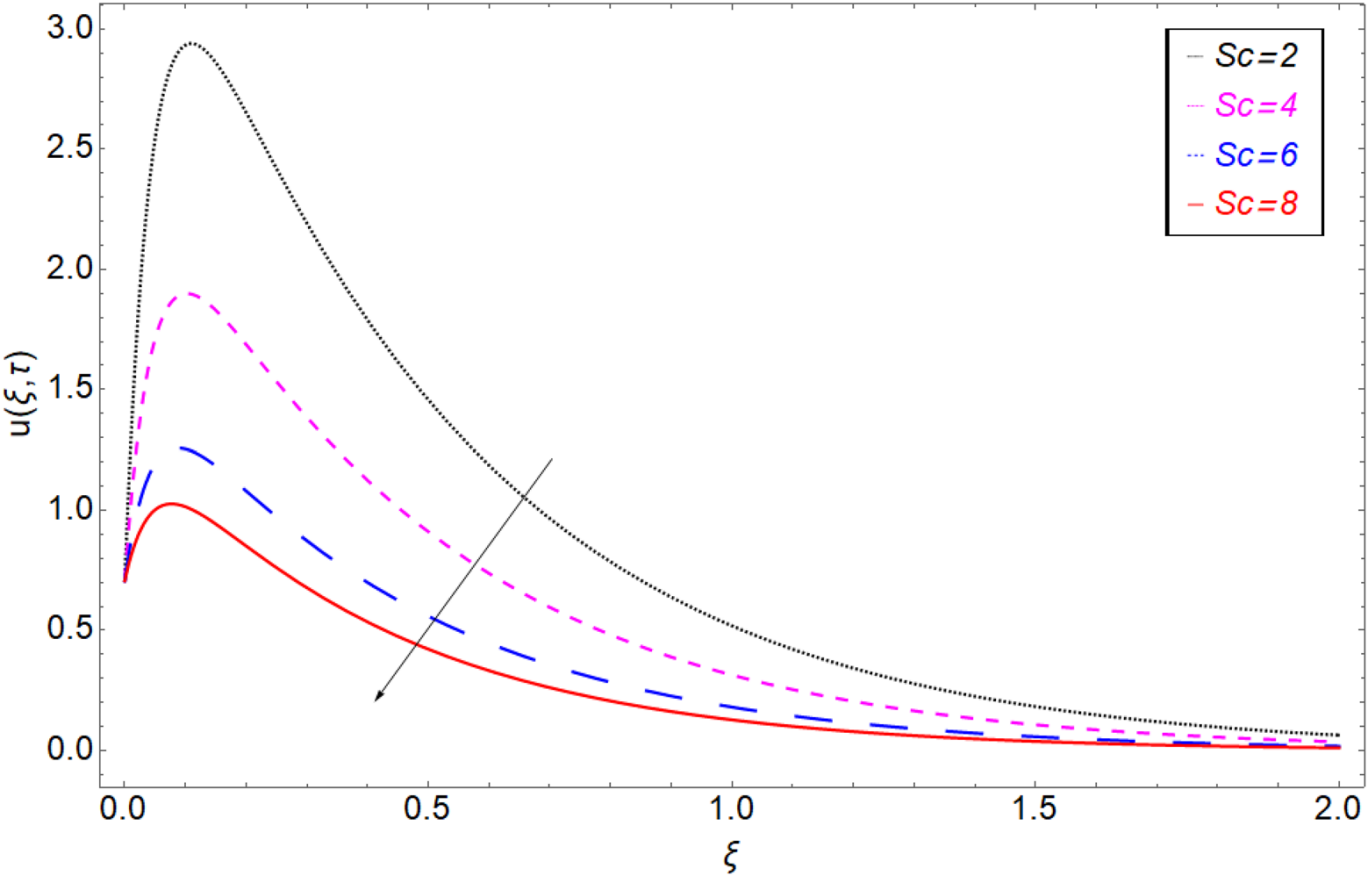
Velocity profile against Schmidth number *Sc.*

**Figure 14:**
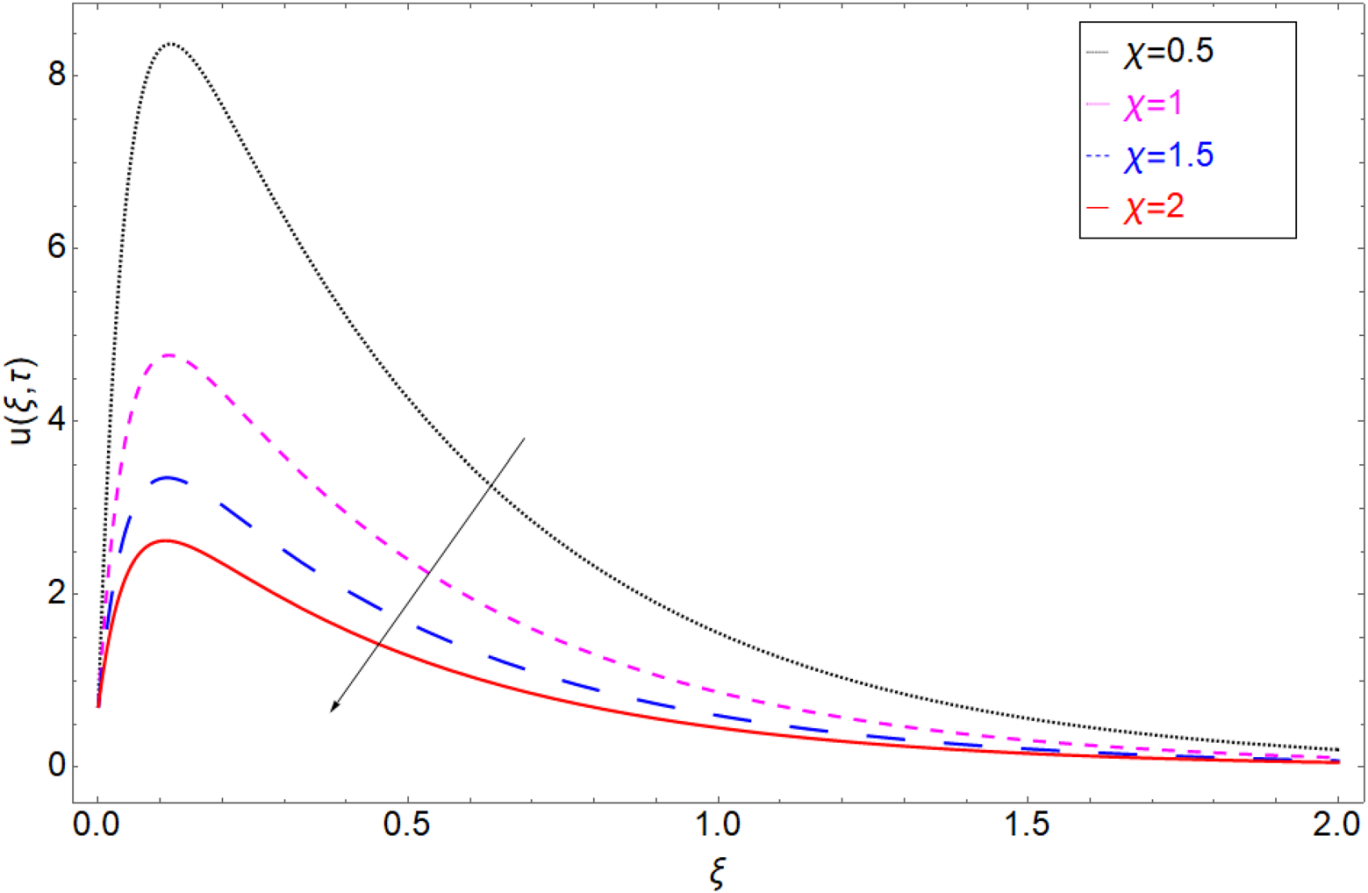
Velocity profile against Heat generation parameter $\chi$.

The impact of the electro-osmotic parameter $Es$ on the fluid motion is portrayed in Fig. 12. The electro-osmotic parameter is in direct contact with the electric double layer (EDL), so as the magnitude of $Es$ rising, the thickness of EDL increase which boost up the fluid in the direction of the fluid motion and hence the fluid velocity accelerate. In order to check the behaviour of fluid motion in response of *Sc,* Fig. 13 has been drawn. Figure 13 shows the decrease in the fluid motion against greater values of *Sc* and it is because of viscous forces that are produce in the fluid due to which the motion of the fluid slow down. Retardation in the velocity field of clay-based water against heat generation parameter $\chi$ has been reported in Fig. 14. As the value of $\chi$ increase the momentum boundary layer thickness decrease due to which retardation in the fluid has been observed.

**Figure 15:**
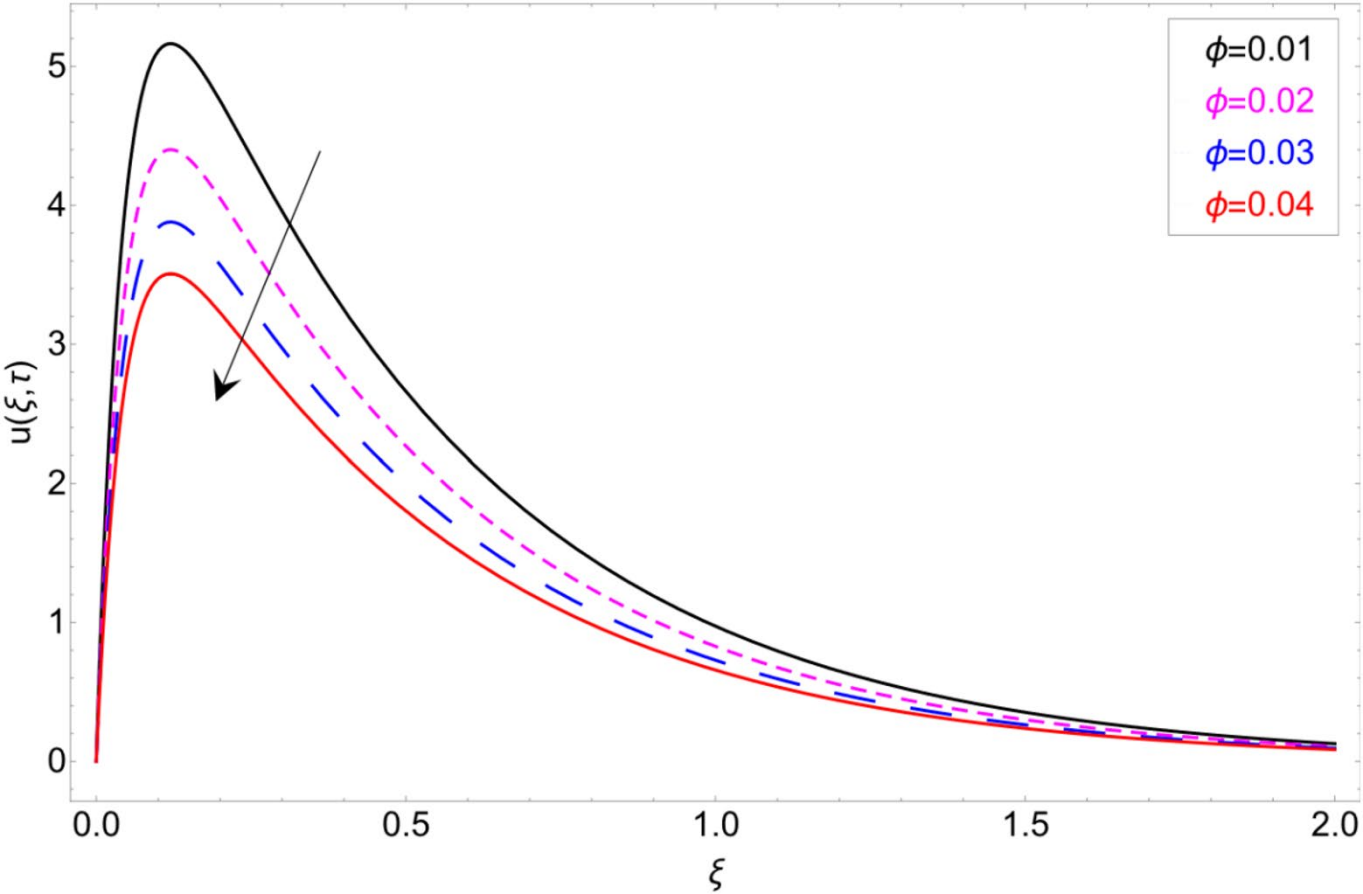
Velocity profile against volume fraction $\phi$.

**Figure 16:**
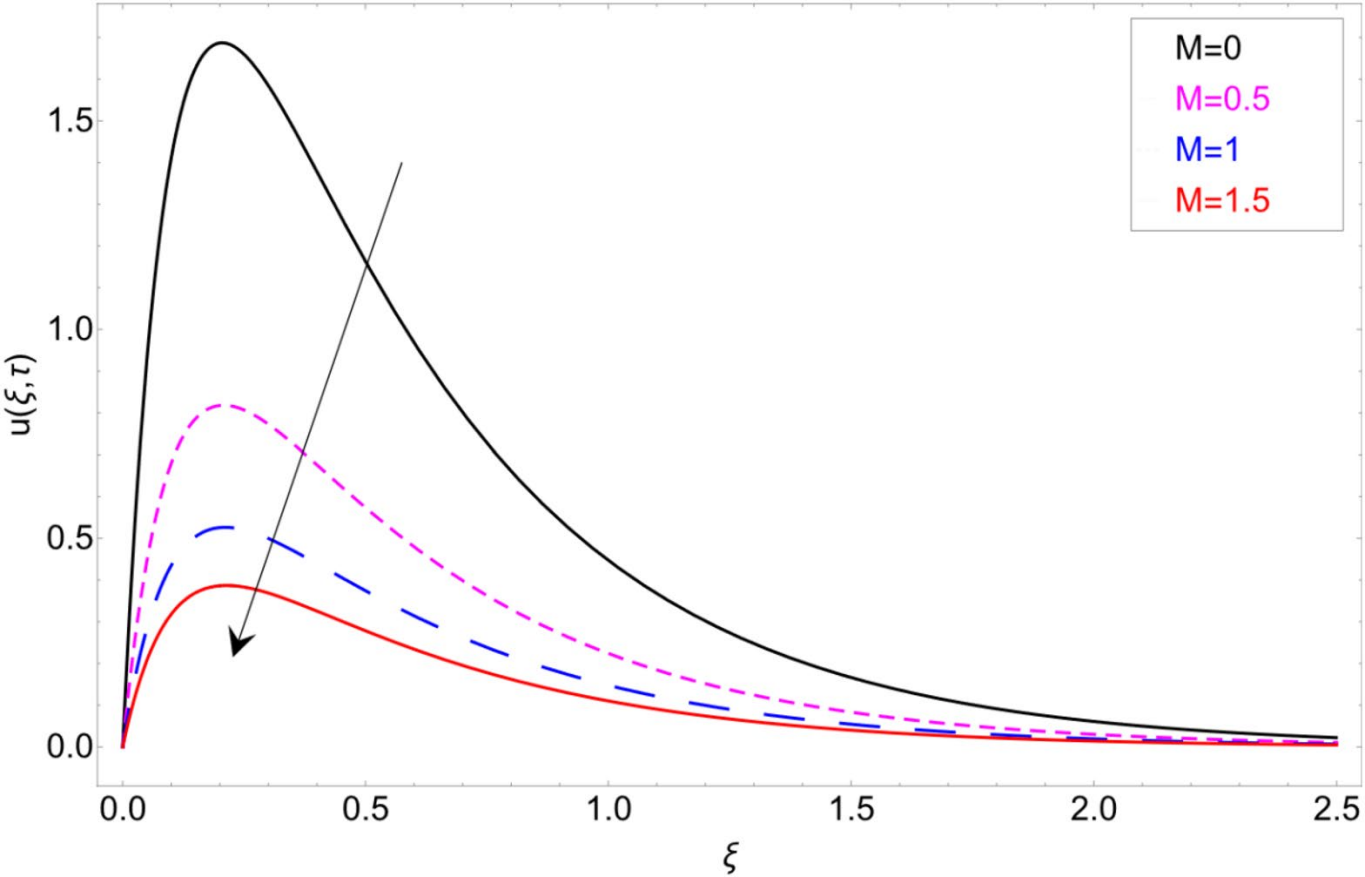
Velocity profile against Magnetic parameter *M.*

Figure 15 shows the behaviour of fluid motion in response to $\phi$ (volume fraction). The density of clay nanomaterials is much higher than base fluid, so the inclusion of clay nanoparticles in the base fluid enhances the average density of nanofluid, which results in the reduction of fluid velocity. The impact of the magnetic strength *M* on fluid motion is discussed in Fig. 16. Physically, magnetic strength generates the Lorentz force, which provide resistance to flow field, that’ why such scenario has been perceived in Fig. 16.

**Figure 17:**
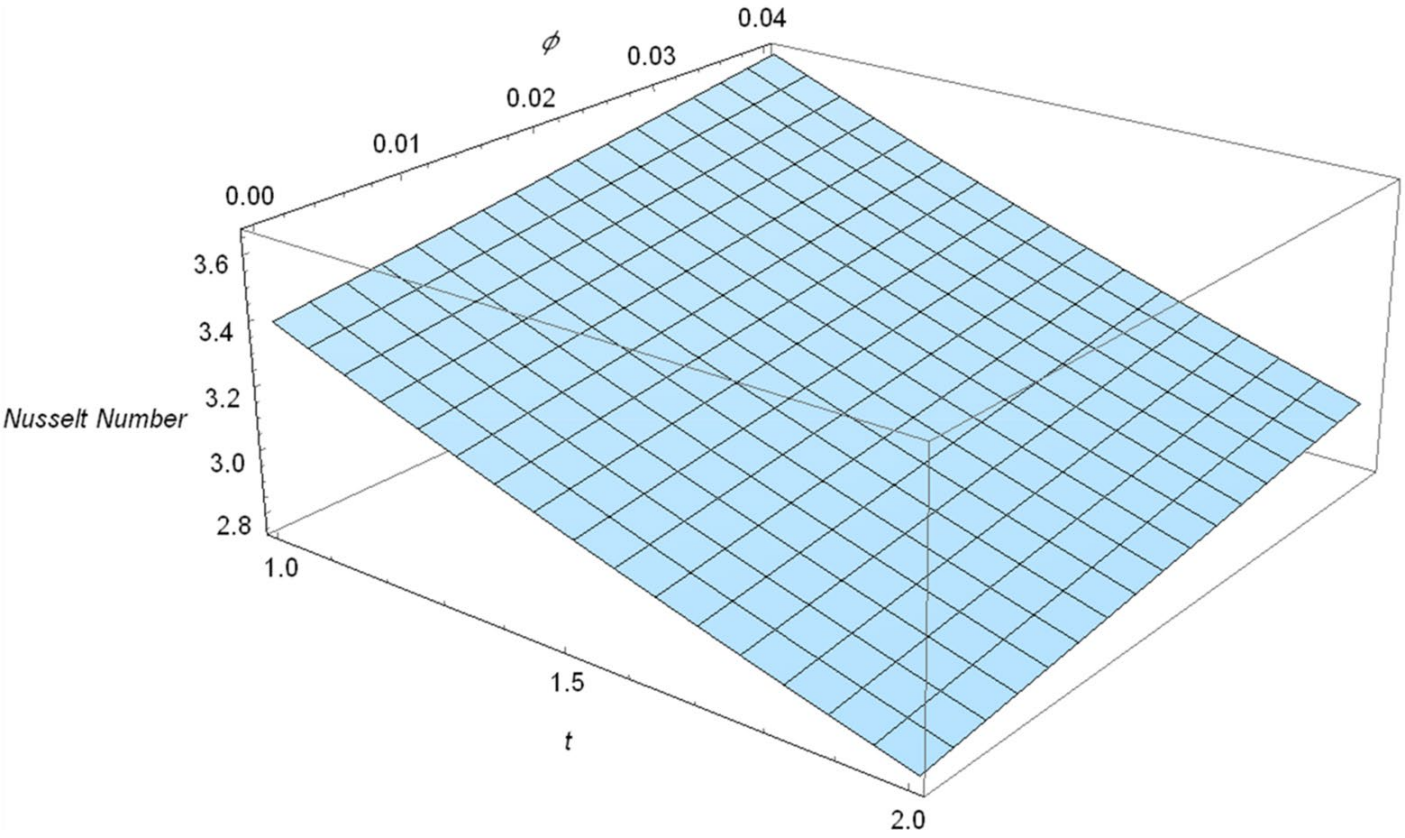
Nusselt number Nu in response of volume fraction $\phi$.

Table 1 revealed the thermo-mechanical characteristics of regular fluid and nanoparticles. Variation in the rate of heat transfer in response of the magnitude of clay nanoparticles is calculated from the obtained exact solution and presented in Table 2. From Table 2 it is very clear that when the volume fraction $\phi$ gradually increases from 0.00 to 0.04 that rate of heat transfer enhances to 11.830% which consequently increases the efficiency of the drilling nanofluid. The same trend is also shown in graphical form in Fig. 17. Change in the rate of mass transfer (Sherwood Number $Sh$) against the volume fraction $\phi$ of the dispersed clay nanoparticles is presented in Table 3. Which shows the rate of mass transfer will enhance 25.5% when the value of $\phi$ reaches to 0.04. This result is very significant infiltration of contaminated water perspective. Due to clay nanoparticle the contaminated water will filtered 25.5% more rapidly than regular water.

**Table 1:** Thermo-mechanical characteristics of regular fluid and nanoparticles^42^.

	$\rho \;\left( {{\text{kg/m}}^{3} } \right)$	$C_{p} \;\left( {\text{J/kgK}} \right)$	$\sigma \;\left( {\Omega {\text{/m}}} \right)$	$K\;\left( {\text{W/mK}} \right)$	$\beta_{C} \times 10^{ - 5} \;\left( {{\text{K}}^{ - 1} } \right)$	$\beta_{T} \times 10^{ - 5} \;\left( {{\text{K}}^{ - 1} } \right)$	$\Pr$
Clay Nanoparticles	6320	531.8	0.2	76.5	1.8	1.80	–
Water	997	4179	0.07197	0.613	0.214	21	6.2

**Table 2:** Variation in the rate of heat transfer in response of volume fraction $\phi$.

$\phi$	Nusselt number	%age enhancement
0.00	3.39642	–
0.01	3.49228	2.822
0.02	3.59100	5.729
0.03	3.69288	8.728
0.04	3.79821	11.830

**Table 3:** Variation in the rate of mass transfer in response of volume fraction $\phi$.

$\phi$	Sherwood NUMBER	%age enhancement
0.00	0.235	–
0.01	0.301	6.6
0.02	0.368	13.3
0.03	0.421	18.6
0.04	0.488	25.6

## Concluding remarks

The electro-osmotic flow of fractionalized Brinkman-type drilling nanofluid based on clay nanoparticles has been examined. The governing equations that governing the fluid motion have been formulated by using relative and appropriate constitutive equations along with physical initial and boundary conditions. By incorporating similarity variables, the dimensional system of equations has been dimensionless and then converted into a time-fractional model by inserting the Caputo–Fabrizio fractional operator. The exact solution has been obtained through the integral transform i.e. Laplace transform technique. The key observations are listed below:

- The fractional parameter $\gamma$ provides more than one profile as compared to the local mathematical model. This outcome elaborates the memory effect in the fluid which is not possible to explain by the local model.
- Velocity profile enhances against *Es Gr* increases while in response of $\phi ,\,\,\beta$ and $M$ decrease.
- Temperature profile shows declination in response of volume fraction $\phi$ of clay nano particles.
- It is interesting to see that the rate of heat transfer of drilling nanofluid is enhanced 11.83% when the magnitude of volume fraction of clay nanoparticles reaches to 0.04. As a result, the drilling nanofluid's effectiveness improves.
- It's noteworthy to note that when the volume fraction of clay nanoparticles reaches 0.04, the rate of mass transfer of drilling nanofluid increases by 25.5%. In terms of contaminated water intrusion, this is a major outcome. Contaminated water will filter 25.5% faster than ordinary water due to clay nanoparticles.
- The current research is important in the process of cleaning polluted water and improving the thermo-physical properties of water, such as thermal conductivity, boiling point, specific heat capacity, and so on, by using clay nanoparticles.

## List of symbols

$u$: Component of velocity
$$t$$: Time
$$y$$: *y*-Axis
$q$: Laplace transform variable
$T$: Temperature of the fluid
$T_{s}$: Temperature of the surrounding
$C$: Concentration of the fluid
$C_{s}$: Concentration of surrounding
$\rho_{nf}$: Density of nanofluid
$\mu_{nf}$: Dynamic viscosity of nanofluid
$\sigma_{nf}$: Electrical conductivity of nanofluid
$\left( {\rho \beta_{T} } \right)_{nf}$: Volumetric thermal expansion of Nanofluid
$\left( {\rho \beta_{C} } \right)_{nf}$: Mass volumetric expansion of nanofluid
$\left( {\rho C_{p} } \right)_{nf}$: Specific heat capacity of nanofluid
$k_{nf}$: Thermal conductivity of nanofluid
$D_{nf}$: Mass diffusivity of nanofluid
$$\beta^{*}$$: Brinkman parameter
$B_{0}^{2}$: Square of the applied magnetic field
$E_{x}$: External electric field
$\rho_{e}$: Total charge density
$$g$$: Gravity
$Q_{0}$: Heat generation term
$\beta = \frac{{\beta^{*} \upsilon }}{{U_{0}^{2} }},\,$: Dimension less Brinkman parameter
$Gr = \frac{{\upsilon \beta_{T} g\left( {T_{P} - T_{s} } \right)}}{{U_{0}^{3} }}$: Thermal Grashof Number
$\,\,Es = \frac{{\nu E_{x} \varepsilon k^{2} \psi_{p} }}{{U_{0}^{3} \rho }}$: Dimensionless electro-osmotic parameter
$\chi = \frac{{Q_{0} \nu }}{{\left( {\rho C_{p} } \right)_{nf} U_{0}^{2} }}$: Dimensionless heat generation parameter
$$Sc = \frac{\nu }{{D_{f} }}$$: Schmidth number
$${\text{Nu}}$$: Nusselt number
$\varepsilon$: Dielectric permittivity of the solvent
$k^{2}$: Debye–Huckel parameter
$\psi_{p}$: Zeta potential at the plate
$$\gamma$$: Fractional order
$$\phi$$: Volume fraction
$\rho_{f}$: Density of the fluid
$\rho_{s}$: Density of the solid nanoparticle
$\mu_{f}$: Dynamic viscosity of the fluid
$\mu_{s}$: Dynamic viscosity of the solid nanoparticle
$k_{f}$: Thermal conductivity of the fluid
$k_{s}$: Thermal conductivity of solid nanoparticles
$\left( {\rho \beta_{T} } \right)_{f}$: Volumetric thermal expansion of the fluid
$\left( {\rho \beta_{T} } \right)_{s}$: Volumetric thermal expansion of solid nanoparticles
$\left( {\rho C_{p} } \right)_{f}$: Specific heat capacity of fluid
$\left( {\rho C_{p} } \right)_{s}$: Specific heat capacity of solid nanoparticles
$\left( {\rho \beta_{C} } \right)_{f}$: Mass volumetric expansion of the fluid
$\left( {\rho \beta_{C} } \right)_{s}$: Mass volumetric expansion of the solid particles
$\sigma_{f}$: Electrical conductivity of fluid
$\sigma_{s}$: Electrical conductivity of solid nanoparticles
$D_{f}$: Mass diffusivity of fluid
$D_{s}$: Mass diffusivity of solid nanoparticles
$H\left( t \right)$: Heaviside step function
$$M = \frac{{\sigma B_{0}^{2} \upsilon }}{{\rho U_{0}^{2} }}$$: Magnetic parameter
$Gm = \frac{{\upsilon \beta_{C} g\left( {C_{P} - C_{s} } \right)}}{{U_{0}^{3} }}$: Mass Grashof number
$$\Pr = \frac{{\mu C_{p} }}{k}$$: Prandtl number
$\alpha = \frac{{k_{1} \nu }}{{U_{0}^{2} }}$: Dimensionless chemical reaction parameter
$$M\left( \gamma \right)$$: Normalization function
$${\text{Sh}}$$: Sherwood number
